# Nanocomposite Membrane Scaffolds for Cell Function Maintaining for Biomedical Purposes

**DOI:** 10.3390/nano11051094

**Published:** 2021-04-23

**Authors:** Monika Drabik, Anna Grzeczkowicz, Paweł Bącal, Angelika Kwiatkowska, Marcin Strawski, Magdalena Antosiak-Iwańska, Beata Kazimierczak, Ewa Godlewska, Ludomira H. Granicka

**Affiliations:** 1 Nalecz Institute of Biocybernetics and Biomedical Engineering, Polish Academy of Sciences, 01-224 Warsaw, Poland; mdrabik@ibib.waw.pl (M.D.); agrzeczkowicz@ibib.waw.pl (A.G.); pawel.bacal@ibib.waw.pl (P.B.); ankwiatkowska@op.pl (A.K.); magdalena.antosiak@ibib.waw.pl (M.A.-I.); beata.kazimierczak@ibib.waw.pl (B.K.); ewa.godlewska@ibib.waw.pl (E.G.); 2 Laboratory of Electrochemistry, Faculty of Chemistry, Warsaw University, 00-927 Warsaw, Poland; marcin@chem.uw.edu.pl

**Keywords:** nanocomposite membrane scaffolds, metal nanoparticles, transport, human fibroblasts, biological characterization, nanotechnology, regenerative medicine

## Abstract

Nanocomposite multilayered membrane coatings have been widely used experimentally to enhance biomedical materials surfaces. By the selection of reliable components, such systems are functionalized to be adjusted to specific purposes. As metal nanoparticles can reduce bacterial cell adhesion, the idea of using gold and silver nanoparticles of unique antimicrobial properties within membrane structure is outstandingly interesting considering dressings facilitating wound healing. The study was aimed to explore the interface between eukaryotic cells and wound dressing materials containing various nanoelements. The proposed systems are based on polyethyleneimine and hydroxyapatite thin layers incorporating metallic nanoparticles (silver or gold). To examine the structure of designed materials scanning electron and transmission electron microscopies were applied. Moreover, Fourier-transform infrared and energy-dispersive X-ray spectroscopies were used. Additionally, water contact angles of the designed membranes and their transport properties were estimated. The functioning of human fibroblasts was examined via flow cytometry to assess the biocompatibility of developed shells in the aspect of their cytotoxicity. The results indicated that designed nanocomposite membrane scaffolds support eukaryotic cells’ functioning, confirming that the elaborated systems might be recommended as wound healing materials.

## 1. Introduction

Wound healing belongs to the complex multi-phase processes of dynamic characteristics. The tissue’s efficient regeneration depends on the number of factors among which the pathological circumstances, environmental conditions and dressing material can be enumerated [1,2,3,4]. For those reasons, it should not be surprised that the appropriate fitting of wound dressing material to the wound increases therapy effectiveness, facilitates healing and reduces the risk of potential infection for both acute and chronic wound types. Furthermore, the application of personalized wound dressing can accelerate treatment [5]. Nevertheless, it is expected that each material aimed at biomedical usage fulfills a list of restrictive requirements. Thereby, comprehensive studies must be conducted to obtain the material matching the appropriate biological and physicochemical characteristics.

The number of natural and synthetic components involving metallic NPs have been tested for their usefulness and cooperation with the cells [6,7,8,9,10,11,12,13]. However, the concept of applying metal nanoparticles as a part of the wound dressing seems to be incredibly engaging, considering the increase of multidrug resistance in microorganisms. Undoubtedly, the development of antibiotics, antivirals and antimalarials are some of modern medicine’s most remarkable successes [14]. Nevertheless, time with these drugs is running out. Antimicrobial resistance—the ability of bacteria, parasites, viruses and fungi to resist these medicines leading to the inability to prevent infections—could seriously compromise medical procedures [15,16]. To counter this problem, the researchers perform intensive studies worldwide to develop new materials or improve existing ones by involving nanocomposites [17,18,19,20,21,22,23]. Due to their unique properties allowing for reducing bacterial cell adhesion, metallic nanoparticles can serve as effective antimicrobials. Thus, they can decrease the risk of infections associated with wound healing. Interestingly, the synthesis of NPs and their surface’s functionalization allows achieving the precise shape, biocompatibility and disparity [24,25], which is also a vast advantage. Moreover, the interaction and multifactorial connections of metal-based NPs with biotic systems are of great importance. Nonetheless, nanoparticles in high concentrations can cause toxic effects on eukaryotic cells [26,27]. Therefore, it is essential to design bacteriostatic materials containing NPs to sustain eukaryotic cells’ functioning. The idea of impregnated the antibacterial nano-agents in modern wound dressing materials was broadly described by many authors previously [28,29,30,31,32,33]. However, only the development of the platforms for the safe handling of nanocomposites can realize nanoelements’ full potential. Moreover, it should not be forgotten that the interactions between cells and nanoparticles at the molecular level still require further studies, especially in the field of cytotoxicity.

Modern wound dressing systems are usually based on hydrogels, which provide the appropriate biocompatibility and maintain a moist healing environment. On the other hand, they show low mechanical strength and poor durability, restricting their usage [34,35,36]. The main challenge is to design a composite material allowing to take advantage of various components’ benefits, improving hydrogels’ physicochemical properties [37], increasing the effectiveness of wound healing while reducing the potential side effects of long-term therapy. The nanocomposite multilayered membrane coatings’ surface functionalization is one of the most interesting, applied approaches because it provides high efficiency and low production costs [6,38]. Nanocomposite multilayered membrane coatings can be easily enhanced by the agents supporting tissue re-modeling like growth factors or antimicrobial agents.

The study was aimed to explore the interface between eukaryotic cells and wound dressing materials containing various nanoelements. Elucidating the influence of nano-functionalized systems on cell functioning may have supported the process of rational designing of materials for tissue regeneration. The study’s purpose was to determine if there were differences in systems’ performance based on miscellaneous nanocomposite membrane scaffolds. We decided to construct polyethyleneimine-hydroxyapatite platforms incorporating metallic nanoparticles (silver or gold) playing bacteriostatic agents’ role. The presence of the inorganic mineral—hydroxyapatite, commonly used as a scaffolding material [39]—had to facilitate wound healing by stimulating collagen production [40]. Moreover, to improve the resulting bandage’s biocompatibility, we employed polyelectrolyte membrane shells as a matrix to hold the remaining nanocomposites. Kong and coworkers previously proposed the drug-delivery system based on similar compounds. The group designed the functionalized porous hydroxyapatite (HAP) nanoparticle-based nanocarriers. The system was built of polyethyleneimine (PEI) coating and outer hyaluronic acid (HA) layer (HAP–PEI–HA) [41]. Our platform’s uniqueness is associated with applying the polyelectrolyte coatings of nano-size thickness; such films can efficiently serve as individual systems and as part of multi-layer bandages aimed to release active factors over time. To achieve the optimal results, we developed the coatings based on polyethyleneimine (PEI), known for its excellent properties as a carrier, broadly used in drug and gene delivery systems dedicated to regenerative medicine, especially wound healing [42,43,44,45]. We designed the platforms of polyethyleneimine membranes modified/unmodified with hydroxyapatite incorporating gold or silver nanoparticles to sustain immobilized eukaryotic cells’ functioning. Cell immobilization provides to limits the free movement of cells without delimiting the access to nutrients and the outflow of waste products. Immobilization on the polyelectrolyte membrane’s surface occurs by adhesion. The process in which the carrier’s ionic interactions with a high density of positive charges and numerous negative charges of cell membranes play the leading role [46]. We perform several experiments using microscopic and spectroscopic techniques to testify the constructed membranes. Furthermore, we evaluated coatings’ transport characteristics. Additionally, to assess the biocompatibility of developed membrane platforms in the aspect of their cytotoxicity, we assessed the function of cells in vitro. The human dermal fibroblasts were used as model cells for immobilization within the designed nano-coatings during these studies.

## 2. Materials and Methods

### 2.1. Materials

Reagents: poly(ethyleneimine), branched, Mn ~60,000, Mw 750,000, analytical standard, 50% (*w*/*v*) in H_2_O (Sigma-Aldrich, München, Germany); silver nanoparticles, 100 ppm, of 10 nm size, stabilized with sodium citrate, in 0.01% Tween 20 (University Technology Transfer Centre of the University of Warsaw (UTTC UW): Bell Synthesis, Poland, EU); golden nanoparticles, 100 ppm, of 10 nm size, stabilized with sodium citrate, in 0.01% Tween 20 (UTTC UW: Bell Synthesis, Warsaw, Poland); hydroxyapatite, aqueous paste, particle size < 50 nm, 30 wt.%, spec. surface area ≥ 80 m^2^/g (Sigma-Aldrich, München, Germany); propidium iodide, ≥94.0% (HPLC) (Sigma-Aldrich, München, Germany); trypsin EDTA Solution C (0.5%), EDTA 0.2% (10X) (Biological Industries, Israel); phosphate-buffered saline (PBS) (Biomed Lublin, Poland, UE); water MilliQ.

Media: Fibroblast Growth Medium (FGM) (Sigma-Aldrich, München, Germany).

Cells: Cell line of Human Dermal Fibroblasts (HDF) (Sigma-Aldrich, München, Germany).

### 2.2. Methods

#### 2.2.1. Membrane Preparation

The membrane scaffolds were prepared based on polyethyleneimine solution at a 1 mg/mL concentration in PBS (PEI). To obtain the membranes:

1. polyethyleneimine with AuNPs composite (PEI-Au)—20 ppm AuNPs solution in PBS was added to PEI at a 1:1 ratio and subsequently stirred for 4 h, room temperature.
2. polyethyleneimine with AgNPs composite (PEI-Ag)—20 ppm AgNPS solution in PBS was added to PEI at a 1:1 ratio and subsequently stirred for 4 h, room temperature.
3. the bilayer of polyethyleneimine incorporating AuNPs and hydroxyapatite (PEI-Au|HAP)—the HAP layer of 0.3% hydroxyapatite solution in PBS was deposited on a PEI-Au membrane layer.
4. the bilayer of polyethyleneimine incorporating AgNPs and hydroxyapatite (PEI-Ag|HAP)—the HAP layer of 0.3% hydroxyapatite solution in PBS was deposited on a PEI-Ag membrane layer.
5. built of the hydroxyapatite mixed with polyethyleneimine incorporating AuNPs (PEI-Au-HAP)—the polyethyleneimine with AuNPs solution (PEI-Au) was added to 0.3% hydroxyapatite solution in PBS at a 1:1 ratio and subsequently stirred for 4 min, room temperature.
6. built of the hydroxyapatite mixed with polyethyleneimine incorporating AgNPs (PEI-Ag-HAP)—the polyethyleneimine with AgNPs solution (PEI-Ag) was added to 0.3% hydroxyapatite solution in PBS at a 1:1 ratio and subsequently stirred for 4 min, room temperature.

Finally, the concentration of nanoparticles in the membranes involving AgNPs or AuNPs was 10 ppm. HAP concentration was 0.15% in PEI-Ag-HAP and PEI-Au-HAP membranes.

**Figure 1:**
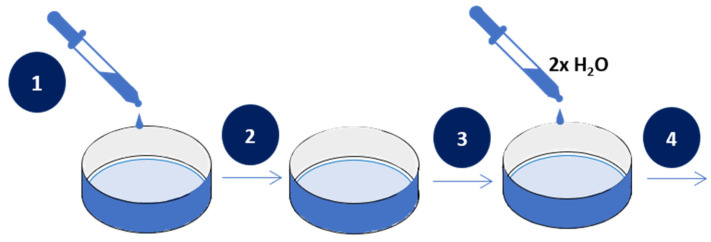
The procedure of membrane deposition. The membrane was poured onto the coverslips (**1**). Then, the membrane dries overnight at room temperature (**2**). The coverslips were washed twice with deionized water (**3**) and transferred to the culture wells (**4**).

The membrane layers were deposited on the support—sterile coverslips. The membrane was poured onto the coverslips, then allowed to dry overnight at room temperature. Next, the coverslips were washed twice with deionized water and, after drying, transferred to the culture wells (put at the bottom of a culture well). The procedure of membrane deposition was present in Figure 1.

#### 2.2.2. Wettability and Work of Adhesion Evaluation

The wettability of polyelectrolyte membranes was studied in a surface energy analyzer Phoenix 150 (Surface Electro-Optics Haas, EU). Before the analysis, the membrane layers were deposited on the glass coverslips; then, the slips were washed and dried consecutively at room temperature for 2 h. We applied deionized water as a probe for surface-free energy evaluation. After the water droplet forming on the studied membrane surface, the contact angle was monitored by a magnifying camera using dedicated software-SEO Software-IMAGE XP. The images were analyzed automatically by software algorithm analysis using a wave function. The measurements were performed at room temperature.

#### 2.2.3. Assessment of the Transport Properties of the Membranes

The alginate cores prepared of 1.5% alginate solution in 0.1 M NaCl coated with the evaluated membranes were used to estimate the membrane transport properties. The alginate cores served as a control. Diffusive permeability was evaluated using a thermodynamic description of diffusive mass transport across a homogenous membrane (Fick’s law) and a two-compartment model [47]. Dextrans of molecular weight of 70 kDa and 150 kDa was used as the model particle in these studies.

#### 2.2.4. Cell Culture

Human Dermal Fibroblast (HDF) cells were maintained under standard culture conditions (5% CO_2_, 37 °C) in Fibroblast Growth Medium. Cells were grown to approximately 80% confluency. The medium was then removed from the culture bottles, whereas the cells were washed with PBS without calcium and magnesium ions and trypsinized with trypsin with EDTA. 

The HDF cells were placed on tested membranes at a density of 3 × 10^3^ cells per well (1.6 × 10^3^/cm^2^). As a negative control, the cells cultured in the presence of support without deposited membranes were applied. Cell viability was assessed after 1, 3 and 7 days of culture in a cytochemical reaction with propidium iodide in a flow cytometer. 

#### 2.2.5. Fluorescent Staining

After 1, 3 and 7 days of culture of cells immobilized within scaffolds, the samples were fixed with 4% paraformaldehyde. After that, the cell membranes were permeabilized using TRITON X100 detergent to provide the dye’s penetration into the individual cells. Then, the fluorescent staining was performed by (1) the addition of fluorochrome-conjugated phalloidin, staining F-actin; (2) the addition of DAPI solution. Phalloidin directly binding to filamentous actin (F-actin) is a toxin isolated from the fungus Phylum Amanita (Amanita phalloides) typically found in significant quantities in fibroblasts. DAPI is an explicitly staining DNA fluorochrome, which allows the emittance of blue light’s cell nuclei under UV light. 

Blue DAPI fluorescence (λ = 460 ÷ 500 nm) and red Phalloidin fluorescence (λ = 570 nm) were observed. Observations and photographs were taken using an Olympus IX70 fluorescence microscope.

#### 2.2.6. Flow Cytometry

The eukaryotic cells’ presence was assessed using a Canto II flow cytometer (Becton Dickinson Immunocytochemistry Systems, Franklin Lake, NY, USA). The obtained results were processed by the FACS Diva software system (Becton Dickinson, Franklin Lake, NY, USA). Evaluated objects were separated from other events based on the light scattering characteristics. 

#### 2.2.7. Scanning Electron Microscopy Evaluation

The systems of cells immobilized within membrane shells after 1, 3 and 7 days of culture were fixed with 2.5% glutaraldehyde. Then, so prepared samples were rinsed several times with water and placed in 75% ethanol for 15 min. The procedure was repeated. The next stage was a 15 min incubation of samples in 99.8% ethanol. The samples were then dried and placed on microscope tables.

Scanning Electron Microscopy (SEM) examinations of the samples were performed using TM-1000 (Hitachi, Tokyo, Japan) scanning electron microscope.

#### 2.2.8. Transmission Electron Microscopy Evaluation

We perform Transmission Electron Microscopy (TEM) analysis of the designed membranes on the FEI Talos F200X transmission microscope at 200 kV (The Thermo Scientific, Waltham, MA, USA). The measurements were conducted in TEM and STEM mode using high-angle annular dark-field imaging. The energy-dispersive X-ray spectroscopy (EDX) on a Brucker BD4 instrument was used to detect the metallic nanoparticles and their mapping. Wherein TEM samples were deposited on a copper grid coated with a carbon holey film.

#### 2.2.9. Atomic Forces Microscopy Evaluation

To examine the surface morphology of the prepared samples, Atomic Forces Microscopy (AFM) was used.

To evaluate the interaction force between the polyelectrolyte layers, we applied the force spectroscopy technique. The silicon cantilever with the SQube (a borosilicate glass colloidal particle sphere of a 10 µm diameter) was used for surface forces acquisition. Before each experiment, we determined a cantilever spring constant value through the ThermalTune method. Forces were measured after 5 s of interaction, wherein the maximum load force was set up to 20 nN. The measurements were performed under in situ conditions (0.15 M NaCl).

To determine the work of adhesion, integration of force-distance dependencies was employed. The calculations were made following the formula: $W_{ad}=\int F_{ad}dz$; where $F_{ad}$ refers to adhesion force and *z* corresponds to the distance of the sphere from a surface.

Atomic Forces Microscopy examinations of the samples were performed using MultiMode 8 (Bruker, Billerica, MA, USA) atomic forces microscope. The force–distant curves were acquired in Nanoscope 7.30 software and then analyzed with Origin 8.50 (OriginLab).

#### 2.2.10. Fourier-Transform Infrared Spectroscopy Analysis

The membranes were analyzed using infrared Fourier-transform infrared spectroscopy (FTIR) using a Digilab Excalibur FTS 3000 Mx spectrometer (Bio-Rad, Hercules, CA, USA) equipped with a reusable ZnSe crystal re-amplifier and a transmission adapter.

#### 2.2.11. Statistical Analysis

The mean values and standard deviations and the significance of difference were calculated in the Statistica 7.1 software. Values where *p* < 0.05 were assumed to be significant.

## 3. Results and Discussion

### 3.1. Characterization of Nanocomposite Membrane Scaffolds

#### 3.1.1. Water Contact Angle Measurements

**Figure 2:**
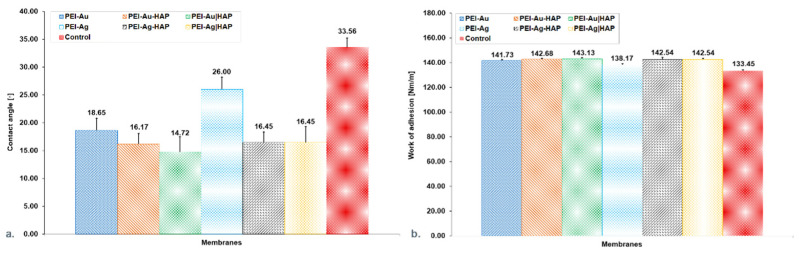
(**a**)—The water contact angle of the composite membranes. (**b**)—Work of adhesion of the composite membranes. Key to symbols: I. Membranes with AgNPs: polyethyleneimine membrane with AgNPs (PEI-Ag); bilayer consisting of polyethyleneimine with AgNPs and hydroxyapatite (PEI-Ag|HAP) and membrane build of polyethyleneimine with AgNPs and HAP complex (PEI-Ag-HAP). II. Membranes with AuNPs: polyethyleneimine membrane with AuNPs (PEI-Au); bilayer consist of polyethyleneimine with AuNPs and hydroxyapatite (PEI-Au|HAP) and membrane build of polyethyleneimine with AuNPs and HAP complex (PEI-Au-HAP). The values are presented as mean ± SD.

The material analysis in terms of wettability (hydrophilicity or hydrophobicity) assesses its suitability for biomedical applications. We tested membranes (1) based on polyethyleneimine incorporating silver or gold nanoparticles (PEI-Ag or PEI-Au); (2) built of hydroxyapatite mixed with polyethyleneimine containing silver or gold nanoparticles (PEI-Ag-HAP or PEI-Au-HAP); (3) a bilayer of hydroxyapatite and polyethyleneimine with silver or gold nanoparticles (PEI-Ag|HAP or PEI-Au|HAP). Obtained results allow stating that there were no significant differences in the contact angles for the membranes involving Au and incorporating Ag and HAP (PEI-Au|HAP; PEI-Ag-HAP). All membranes are hydrophilic (Figure 2a). Similar conclusions can be drawn from the work of adhesion values (Figure 2b).

**Figure 3:**
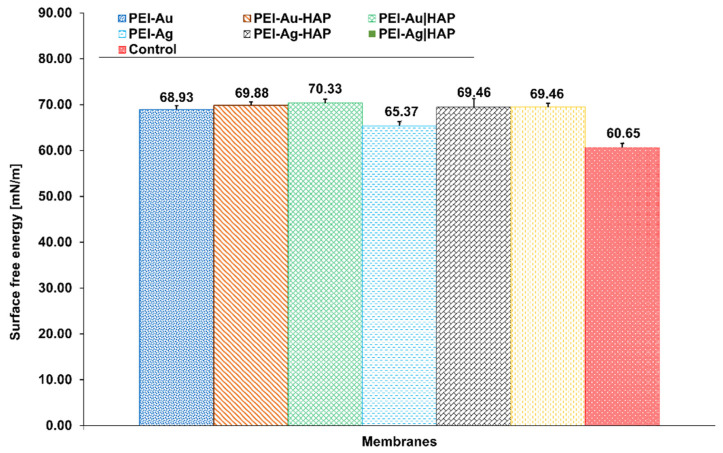
Surface free energy of the composite membranes. Key to symbols: I. Membranes with AgNPs: polyethyleneimine membrane with AgNPs (PEI-Ag); bilayer consisting of polyethyleneimine with incorporated AgNPs and hydroxyapatite (PEI-Ag|HAP); polyethyleneimine with AgNPs and HAP complex (PEI-Ag-HAP). II. Membranes with AuNPs: polyethyleneimine with AuNPs (PEI-Au); bilayer consisting of polyethyleneimine with incorporated AuNPs and hydroxyapatite (PEI-Au|HAP); polyethyleneimine with AuNPs and HAP complex (PEI-Au-HAP). The values are presented as mean ± SD.

Surface energy at the level of 20–30 nm/m is called critical energy, i.e., it is characterized by athrombogenicity and has been defined as a hypothetical biocompatibility zone understood as a zone of minimal cell adhesion. Materials with surface energy above 40 nm/m promote cell adhesion [48]. Obtained values of the surface energy of all constructed membranes (PEI-Ag, PEI-Au, PEI-Ag-HAP, PEI-Au-HAP, PEI-Ag|HAP, PEI-Au|HAP) remained in the range of the value of biomaterials willingly populated by cells (Figure 3) [48].

#### 3.1.2. Assessment of the Transport Properties of the Membranes

**Figure 4:**
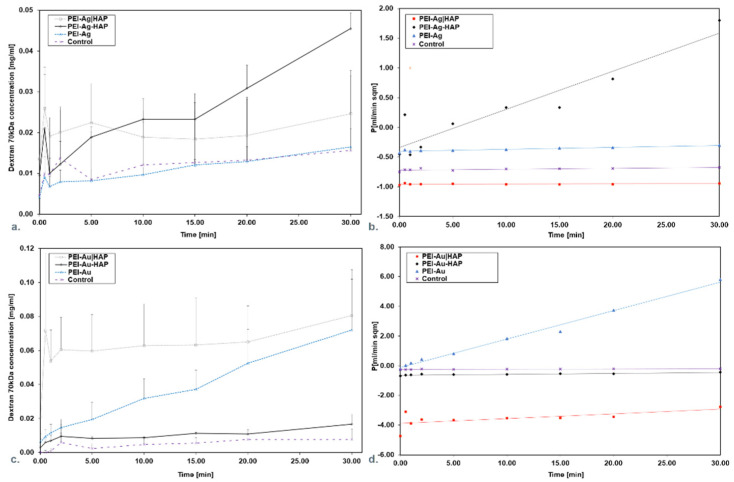
Dextran 70 concentration in the function of time during permeation through the membrane scaffolds containing silver (**a**) or gold (**c**) nanoparticles. Membrane permeability and time product during particular time intervals permeation for Dextran 70 through the membrane scaffolds containing silver (**b**) or gold (**d**) nanoparticles. Key to symbols: PEI-Ag: polyethyleneimine with AgNPs; PEI-Ag|HAP: polyethyleneimine with AgNPs—hydroxyapatite; PEI-Ag-HAP: polyethyleneimine with AgNPs and HAP complex. PEI-Au: polyethyleneimine with AuNPs; PEI-Au|HAP: polyethyleneimine with AuNPs—hydroxyapatite; PEI-Au-HAP: polyethyleneimine with AuNPs and HAP complex. The values are presented as mean ± SD.

The control group and PEI-Ag, adsorbed Dextran 70 (Dex 70), allowing for about 20% release related to the initial concentration value of 0.05 mg/mL. On the other hand, PEI-Ag|HAP provides for the release of about 50% as related to the initial concentration value of 0.05 mg/mL (Figure 4a).

This is reflected in keeping permeability (P) at a consistent level (Figure 4b). It can be stated that PEI-Ag functioning as an independent coating—as a monolayer or in bilayers adsorbed Dex 70. It was observed that only PEI-Ag-HAP allows for a 90% release.

Analyzing the membranes involving AuNPs, it was noticed that the control group and PEI-Au-HAP adsorbed Dex 70, allowing for about 25% release as related to the initial concentration value of 0.08 mg/mL for 30 min. On the contrary, PEI-Au|HAP and PEI-Au allowed for the release of about 94% of the initial concentration value (0.08 mg/mL) (Figure 4c). All indicated that PEI-Au functioning in independent coating (monolayer) or in bilayers did not allow for adsorption of more than 6% of Dex70’s initial concentration. It was reflected in keeping P at a consistent level (Figure 4d).

The discrepancies between the Dex70 permeability for the membranes encompassing the PEI-Au or PEI-Ag as a layer might be caused by differences in dextran’s affinity towards applied nanoparticles. The higher permeability for PEI-Au may occur due to the higher affinity of dextrans towards AuNPs. Some authors reported that dextrans are used as stabilizers of colloidal AuNPs solution [49].

**Figure 5:**
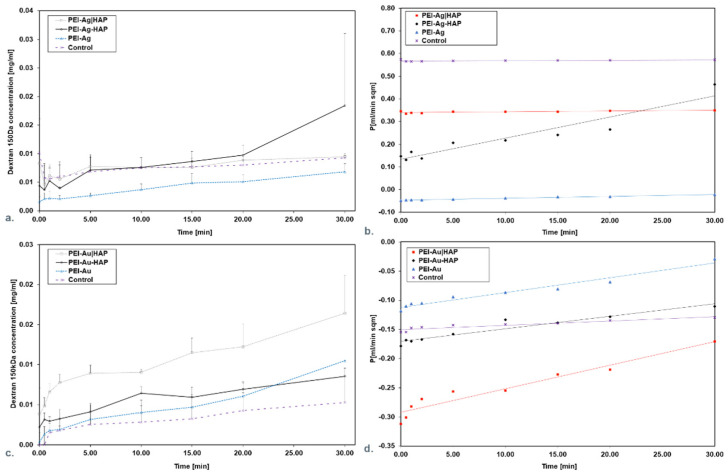
Dextran 150 concentration in the function of time during permeation through the membrane scaffolds containing silver (**a**) or gold (**c**) nanoparticles. Membrane permeability and time product during particular time intervals permeation for Dextran 150 through the membrane scaffolds containing silver (**b**) or gold (**d**) nanoparticles. Key to symbols: PEI-Ag: polyethyleneimine with AgNPs; PEI-Ag|HAP: polyethyleneimine with AgNPs—hydroxyapatite; PEI-Ag-HAP: polyethyleneimine with AgNPs and HAP complex. PEI-Au: polyethyleneimine with AuNPs; PEI-Au|HAP: polyethyleneimine with AuNPs—hydroxyapatite; PEI-Au-HAP: polyethyleneimine with AuNPs and HAP complex. The values are presented as mean ± SD.

The control group and all membranes involving Ag exhibited Dex150 release up to 10% (Figure 5a).

The control group and PEI-Au, PEI-Au-HAP and PEI-Au|HAP allowed the release meanly about 5%, related to the initial concentration. PEI-Au|HAP provided the release of about 10% (Figure 5b). The results indicated the membranes incorporating AgNPs or AuNPs cut-off value at the 150 kDa level. The obtained values are reflected in the P course (Figure 5c,d).

#### 3.1.3. Fourier Transform Infrared Spectroscopy

We assessed Fourier transform infrared spectroscopy (FTIR) signals for polyethyleneimine (PEI), as well as hydroxyapatite (HAP) membranes. Moreover, we analyzed the bilayer build of polyethyleneimine and hydroxyapatite (PEI|HAP). The spectrum was recorded in the range of 4000–400 cm^−1^. The characteristic picks were detected at 3375 cm^−1^ exhibiting N-H stretching vibrations in PEI, 1366 cm^−1^ exhibiting C–CH_3_ presence and 570 cm^−1^ from n4 symmetric P-O stretching vibration of the PO_4_^3−^. Moreover, the characteristic PO_4_^3−^ absorption band was observed at 1631 cm^−1^. After HAP deposition, the pick 3375 cm^−1^ was dislocated to 3325 cm^−1^ in membrane PEI|HAP (Supplementary Material: Figure S1).

#### 3.1.4. Transmission Electron Microscopy Investigations

**Figure 6:**
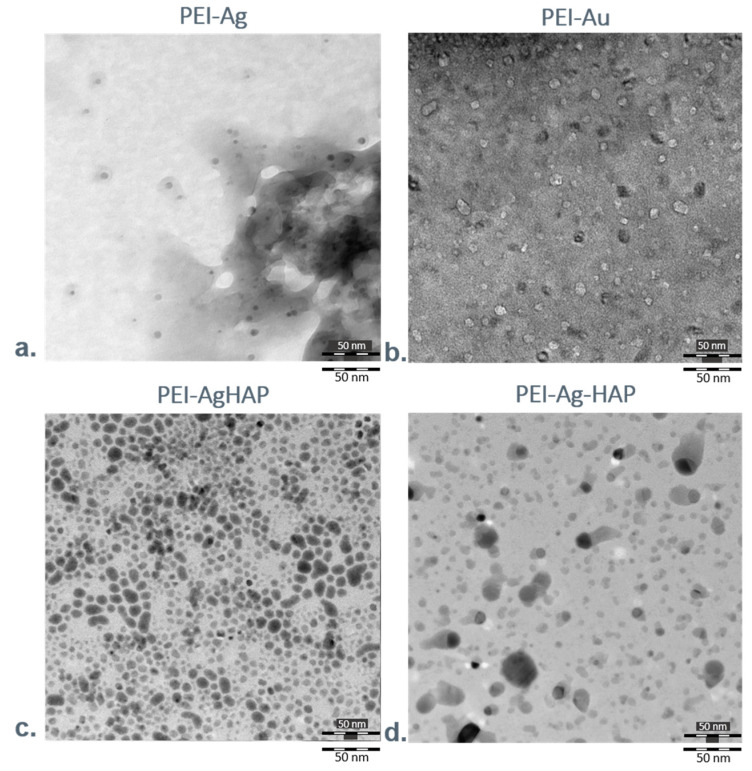
Transmission Electron Microscopy photographs showing the surface of examined membranes. Key to symbols: (**a**) the surface of the polyethyleneimine membrane incorporating AgNPs (PEI-Ag); (**b**) the surface of polyethyleneimine membrane incorporating AuNPs (PEI-Au); (**c**) the surface of polyethyleneimine incorporating AgNPs|hydroxyapatite (PEI-Ag|HAP); (**d**) the surface of hydroxyapatite mixed with polyethyleneimine incorporating AgNPs (PEI-Ag-HAP).

We analyzed surfaces of designed polyelectrolyte membranes using Transmission Electron Microscopy (TEM). Figure 6a,b presents photographs showing surfaces of the films built of polyethyleneimine with silver or gold nanoparticles incorporated within their structure (PEI-Ag or PEI-Au). 

The single, immobilized silver nanoparticles can be observed within the polyelectrolyte layers; they are deposited evenly in membrane material, creating a uniform structure. Conversely, gold nanoparticles formed separate centers positioned within the polyelectrolyte.

Figure 6c,d presents the exemplary photographs of surfaces of the coatings with HAP share. The first image shows the bilayer—built of the polyethyleneimine incorporating AgNPs and hydroxyapatite (PEI-Ag|HAP), whereas the second picture presents the membrane constructed with one layer, consisted of hydroxyapatite mixed with polyethyleneimine incorporating AgNPs (PEI-Ag-HAP). HAP presence does not disturb to distinguish the single silver particles incorporating the polyelectrolyte membrane layer structure.

**Figure 7:**
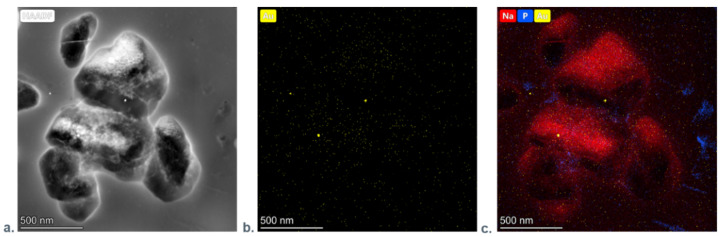
(**a**) STEM image of the PEI-Au-HAP; (**b**) corresponding EDX map of the Au distribution; (**c**) corresponding EDX map of the Na, P, Au distribution.

Since the gold nanoparticles view is overlapped with HAP on TEM’s photographs, we could not confirm their occurrence in membranes with AuNPs using this technique. Thus, to prove gold nanoparticles’ presence in the layers, we applied a high-resolution transmission electron microscopy (HRTEM) coupled with energy dispersive X-ray analysis (EDX). Moreover, we analyzed the nanoelements at the atomic scale by electron diffraction (Figure 7a). The energy-dispersive X-ray mapping technique, which uses a scanning transmission electron microscope (STEM), provides simultaneous nanoparticle visualization together with the corresponding Au distribution map tracing. Figure 7b shows EDX spectra of PEI-Au-HAP membrane: the gold nanoparticles are visible as bright spots. On the studied surfaces, we have also observed the presence of the other elements like Na (being a part of the stabilizer of Au), P (as a substrate of HAP), Cu and C (not presented here (Figure 7c).

EDX spectra of PEI-Au-HAP membrane confirm the presence of the atoms mentioned above (Supplementary Material: Figure S2).

**Figure 8:**
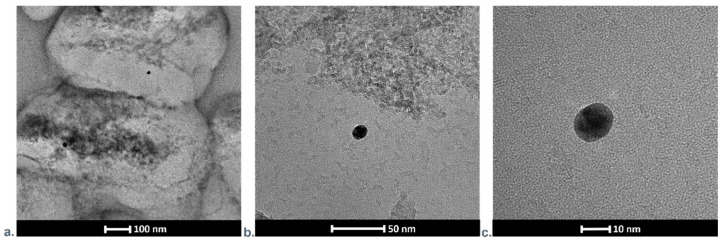
SEM images presenting HAP nanocrystal with adsorbed gold nanoparticles in different magnifications. (**a**) 100 nm, (**b**) 50 nm and (**c**) 10 nm.

We visualized the presence of gold nanoparticles absorbed on hydroxyapatite by scanning transmission electron microscopy. The appropriate photographs in different magnifications are shown in Figure 8a–c. It can be noted that the STEM images indicated that the maximal size of HAP objects is even about ten times higher than the size of elements stated by the manufacturer (<50 nm), which might be caused by its aggregation.

Every membrane was analyzed in three samples/repetitions. The pictures were taken in different sites of the sample (at eight sites). The concentration of nanoparticles in the membranes involving AgNPs or AuNPs was 10 ppm.

#### 3.1.5. Atomic Forces Microscopy Evaluation

**Figure 9:**
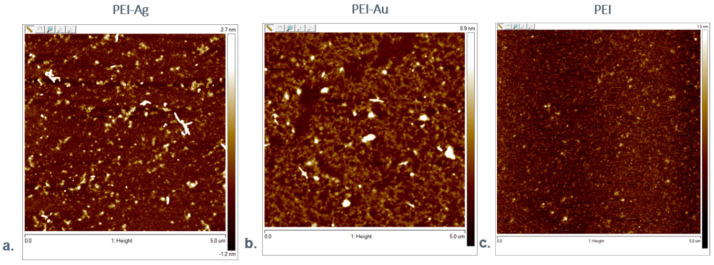
AFM visualization of the layers deposited on the gold mica substrate cover. Key to symbols: (**a**) The surface of the polyethyleneimine membrane with AgNPs incorporating (PEI-Ag); (**b**) the surface of polyethyleneimine membrane with AuNPs incorporating (PEI-Au); (**c**) The surface of the polyethyleneimine membrane.

**Figure 10:**
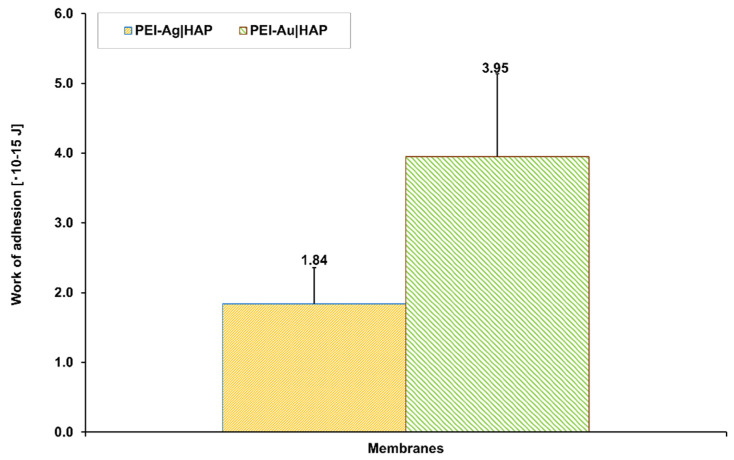
The work of adhesion between the HAP and PEI-Ag or HAP and PEI-Au layers.

Figure 9 shows the visualization of the morphological structure of the designed layers. The samples of PEI, PEI-Ag, PEI-Au were deposited on the gold mica substrate. On the other hand, Figure 10 presents the values of the work of adhesion between HAP and PEI-Ag or PEI-Au layers.

### 3.2. Evaluation of the Functioning of Cells within Different Membranes

#### 3.2.1. Flow Cytometry Analysis

**Figure 11:**
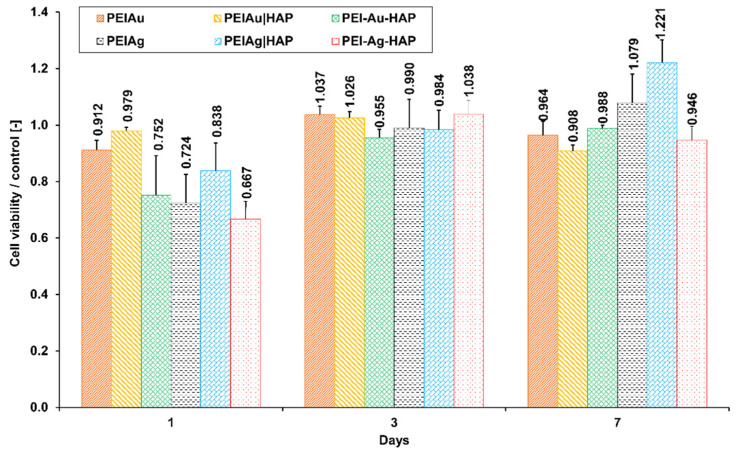
The ratio of the percentage of viable human dermal fibroblasts immobilized within membranes to the control during 7-day culture. Key to the symbols: polyethyleneimine membrane with AgNPs (PEI-Ag), bilayer consisting of polyethyleneimine with incorporated AgNPs and hydroxyapatite (PEI-Ag|HAP); polyethyleneimine with AgNPs and HAP complex (PEI-Ag-HAP); polyethyleneimine with AuNPs (PEI-Au); bilayer consisting of polyethyleneimine with incorporated AuNPs and hydroxyapatite (PEI-Au|HAP); polyethyleneimine with AuNPs and HAP complex (PEI-Au-HAP). The values are presented as mean ± SD.

Figure 11 presents the percentage of viable cells immobilized within the membranes involving AuNPs or AgNPs.

After 7-day culture, no significant difference between PEI-Au and PEI-Au-HAP membranes was observed. The discrepancies in the viability of cells cultured within membranes involving AuNPs did not exceed 8%. There were meanly 95% cells alive.

Similarly, no significant difference between PEI-Ag and PEI-Ag-HAP and PEI-Ag|HAP were observed. However, the viability of cells immobilized within PEI-Ag|HAP was significantly higher than the membranes incorporating AuNPs and compared with PEI-Ag-HAP. Nevertheless, the discrepancies did not exceed about 20%.

Finally, there was no significant difference between the viability of cells immobilized within the membranes built of PEI incorporating AgNPs, functioning as an independent layer in monolayer or bilayer coating.

Gold nanoparticles are generally designed for intracorporeal activity due to the low cytotoxicity towards the organism observed by many authors [25,50,51]. On the other hand, silver nanoparticles are proposed mainly for external usage (e.g., dressings, medical devices, water treatment, decontamination), possibly—in low concentrations—internally, e.g., in implants [50,51,52]. However, when examining the effect of AgNPs and AuNPs on human fibroblast cells, no significant differences in cytotoxicity were found at the NPs concentrations used.

To sum up, it can be stated that applied membranes with metallic NPs did not exert a cytotoxic effect on the HDF cells.

#### 3.2.2. Microscopic Evaluation of Immobilized Cells

**Figure 12:**
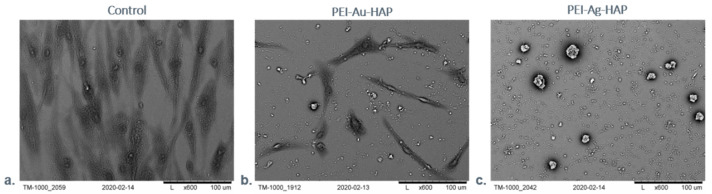
SEM visualization of HDF cells maintained in the absence (control)/presence of the selected, designed membranes after 7-days of the culture. Key to symbols: (**a**) Control (**b**) the membrane build of the hydroxyapatite mixed with polyethyleneimine with AuNPs incorporating (PEI-Au-HAP); (**c**) the membrane build of the hydroxyapatite mixed with polyethyleneimine with AgNPs incorporating (PEI-Ag-HAP). Magnitudes: ×1.0 k.

We performed a scanning electron microscopy (SEM) examination to assess the morphology of the designed scaffolds’ systems with immobilized cells. The exemplary images of such analysis are shown in Figure 12. The cells were grown directly on a slide (without membranes) served as a control.

After seven days of the culture, we observed numerous cells on the surface of films built of the hydroxyapatite mixed with polyethyleneimine incorporating metallic nanoparticles (PEI-Au-HAP and PEI-Ag-HAP). However, cell morphology differed between the membranes. The spindle-shaped cells of fibroblastoid features were present on the PEI-Au-HAP layer’s surface; the cells’ morphology was comparable with the control. On the other hand, on the PEI-Ag-HAP membrane’s surface, the spherical shape cells’ presence was confirmed. The form taken by the cells would indicate that the membranes involving AuNPs facilitating cells to form the shape characteristic for the adhesion phase and allowing for higher adhesion of immobilized cells comparing with the membranes containing AgNPs. Though there was no significant difference in adhesion work between the membranes with HAP content: PEI-Au-HAP and PEI-Ag-HAP, the internalization of HAP by cells might appear, leading to the modification of the membrane surface towards the membrane built mainly of PEI-Au and PEI-Ag, respectively. Thus, the higher value of the work of adhesion of the membranes built of PEI-Au may result in a more spindle shape of cells at the interface with the membrane. However, separate studies of the internalization of HAP NPs at the cellular level would be necessary for confirmation.

The obtained results suggest that the membranes involving AuNPs facilitating cells to form the shape characteristic for the adhesion phase allowed for higher adhesion of immobilized cells comparing with the membranes containing AgNPs. Nevertheless, there was no correlation between immobilized cells’ shape and the cell viability cytometrically assessed.

#### 3.2.3. Fluorescent Staining

The exemplary images of fluorescence microscopic examination of cells maintained in the presence of designed membranes are shown below, wherein the cells grown directly on a slide (without membranes) served as a control.

**Figure 13:**
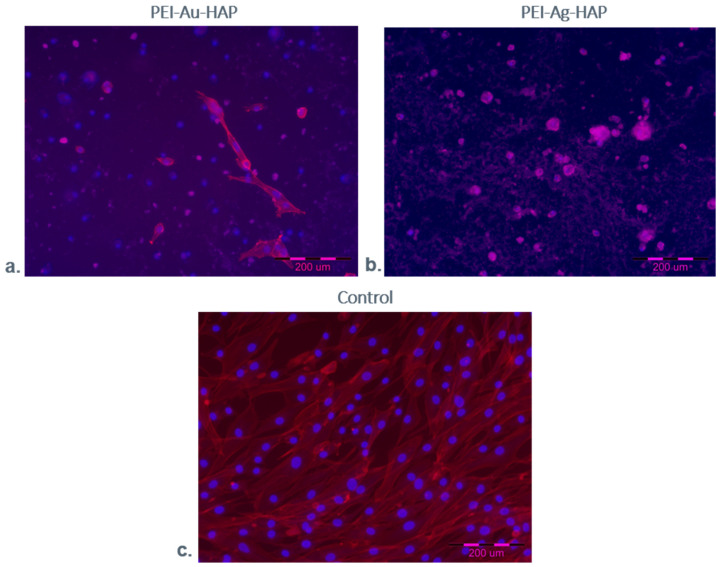
Fluorescence microscopic visualization of HDF cells after 7-day culture within a membrane. Key to symbols: (**a**) PEI-Au-HAP membrane; (**b**) PEI-Ag-HAP membrane; (**c**) Control. Cell nuclei are visualized by staining with DAPI blue fluorescent dye; cell cytoskeleton is stained red with F-Actin.

Figure 13 shows the HDF cells maintained in the absence (control) or the presence of PEI-Ag-HAP or PEI-Au-HAP coatings after seven days of the culture. Cell nuclei are visualized by staining with DAPI blue fluorescent dye; cell cytoskeleton is stained red with F-Actin.

We observed the cells with different morphology for all days of culture. Additionally, the microscopic fluorescence pictures confirmed the cells of spherical shape on the PEI-Ag-HAP membrane. The spindle-shaped cells’ presence, comparable to the control, was visible on the PEI-Au-HAP membrane’s surface. However, the cells of spherical shape were also noticeable.

## 4. Conclusions

We applied the nanocomposite material with metallic nanoparticles (AuNPs or AgNPs) to ensure the material’s bacteriostatic properties and simultaneously maintain its biocompatibility. Some differences were observed between the membranes involving AgNPs or AuNPs concerning their properties and morphology at membrane-immobilized cells interface. The different values of adhesion work were observed between HAP and layers incorporating AgNPs or AuNPs. The cells cultured on the shells involving AgNPs and AuNPs differ in morphology. The more spindle form taken by the cells immobilized on the membranes PEI-Au-HAP involving AuNPs comparing with membrane PEI-Ag-HAP involving AgNPs implies the influence of a higher adhesion work in case PEI-Au-HAP membrane. Such a phenomenon may arise in case of internalization of HAP by immobilized cells what needs further studies.

Nevertheless, the presented data indicated that the designed materials were biocompatible in the aspect of cytotoxicity. The AuNPs or AgNPs involvement at an applied concentration in the scaffold material does not exert a cytotoxic effect on HDF cells.

However, application of AgNPs or AuNPs at concentration 10 ppm incorporated in membranes did not exert a cytotoxic effect on HDF cells, the cytotoxicity of NPs towards biological material still needs further complex examinations in general and in a complex with selected materials that can find biomedical application, in particular, in bandages.

The bacteriostatic membrane with a cut-off at IgG level may be regarded as an element of bandage systems. The designed scaffolding layered shells, especially ensuring physiological shape forming by HDF cells, can be recommended as an appropriate element to sustain eukaryotic cell function in bandage applications.

## Supplementary Materials

The following are available online at https://www.mdpi.com/article/10.3390/nano11051094/s1, Figure S1: Fourier Transform Infrared Spectroscopy spectrum of PEI, HAP, and PEI|HAP membranes. The characteristic picks were detected at 3375 cm^−1^ exhibiting N–H stretching vibrations in PEI, 1366 cm^−1^ exhibiting C–CH_3_ presence, and 570 cm^−1^ from n4 symmetric P–O stretching vibration of the PO_4_^3−^. The characteristic PO_4_^3−^ absorption band was observed at 1631 cm^−1^. After HAP deposition, the pick 3375 cm^−1^ was dislocated to 3325 cm^−1^ in membrane PEI|HAP, Figure S2: EDX spectra of PEI-Au-HAP membrane with Au picks visible.
