# Supplementary material for: Nanocomposite Membrane Scaffolds for Cell Function Maintaining for Biomedical Purposes
Source: Nanomaterials (Basel). 2021 Apr 23;11(5):1094. doi: 10.3390/nano11051094 (PMC8146798; doi:10.3390/nano11051094)
Supplement: Supplementary file 1 [file nanomaterials-11-01094-s001.zip › nanomaterials-1163435-supplementary.pdf]

nanomaterials

Supplementary Materials: Nanocomposite Membrane Scaffolds for Cell Function Maintaining for Biomedical Purposes

Monika Drabik [1], Anna Grzeczkowicz [1], Paweł Bącal [1], Angelika Kwiatkowska [1], Marcin Strawski [2], Magdalena Antosiak-Iwańska [1], Beata Kazimierczak [1], Ewa Godlewska [1] and Ludomira H. Granicka [1,*]

To chapter: 3.1.3. Fourier Transform Infrared Spectroscopy

Fourier-transform infrared spectroscopy (FTIR) signals were assessed. The polyethyleneimine (PEI) and hydroxyapatite (HAP) membranes, as well as bilayer build of polyethyleneimine and hydroxyapatite (PEI|HAP) were analyzed (Figure S1). The spectrum was recorded in the range of 4000–400 cm^{-1}.

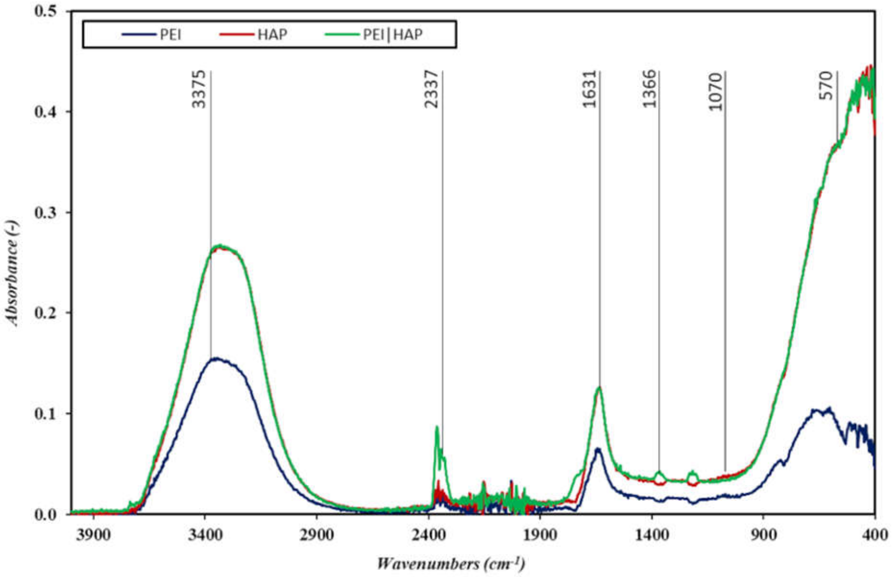

Figure S1. Fourier Transform Infrared Spectroscopy spectrum of PEI, HAP, and PEI|HAP membranes. The characteristic picks were detected at 3375 cm^{-1} exhibiting N–H stretching vibrations in PEI, 1366 cm^{-1} exhibiting C–CH_3 presence, and 570 cm^{-1} from n4 symmetric P–O stretching vibration of the PO_4^{3-}. The characteristic PO_4^{3-} absorption band was observed at 1631 cm^{-1}. After HAP deposition, the pick 3375 cm^{-1} was dislocated to 3325 cm^{-1} in membrane PEI|HAP.

To chapter: 3.1.4. Transmission Electron Microscopy investigations

To confirm gold nanoparticles' presence in the layers, we applied a high-resolution transmission electron microscopy (HRTEM) coupled with energy dispersive X-ray analysis (EDX). EDX spectra of PEI-Au-HAP membrane prove the presence of Au as well as other elements like Na (being a part of the stabilizer of Au), P (as a substrate of HAP), Cu, and C (Figure S2).

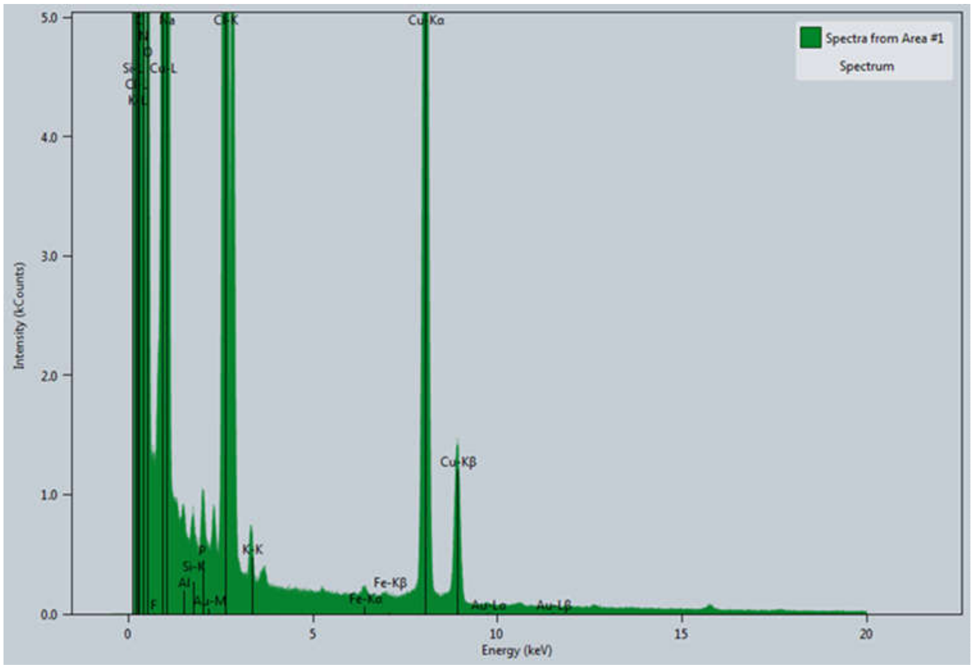

Figure S2. EDX spectra of PEI-Au-HAP membrane with Au picks visible.
